# Experiences of keratoconus patients attending public eye care facilities in South Africa

**DOI:** 10.4102/phcfm.v15i1.3974

**Published:** 2023-06-26

**Authors:** Pheagane M.W. Nkoana, Percy K. Mashige, Vanessa R. Moodley

**Affiliations:** 1 Discipline of Optometry, Faculty of Health Sciences, University of KwaZulu-Natal, Durban, South Africa; 2 Department of Optometry, Faculty of Health Sciences, University of Limpopo, Polokwane, South Africa

**Keywords:** experiences of patients, Capricorn District of Limpopo province, patients with keratoconus, vision-quality of life, keratoconus

## Abstract

**Background:**

Keratoconus (KC) is a progressive condition that usually affects young persons between their first and fourth decades. Myopia and irregular astigmatism are the common presentations which are proceeded by corneal steepening and thinning. Keratoconus is a progressive ectasia of the cornea that presents bilaterally, although often asymmetrical.

**Aim:**

To explore the experiences of KC patients attending public eye care facilities in Capricorn District of Limpopo province.

**Setting:**

Capricorn District, Limpopo province, South Africa.

**Methods:**

Descriptive qualitative phenomenology approach was used in the study to explore the lived experiences of patients diagnosed with KC, attending public eye care facilities. Purposive sampling was used to select 16 patients who were diagnosed with KC and referred for contact lens fitting. Data were collected through face-to-face, one-on-one interviews.

**Results:**

Patients reported to have developed gradual vision loss which worsened when they grew older. There was lack of knowledge of KC amongst patients and this was exacerbated by limited health literacy and vocabulary barriers. Patients had difficulty performing daily activities where some had dropped out of school while others worked at a slower pace and reduced working distances.

**Conclusion:**

Patients with KC received inadequate information on their condition due to limited health literacy and vocabulary barriers. Programmes to promote practitioner and patient education are required to improve the perceived service level provided to KC patients.

**Contribution:**

The findings of this study will assist to improve the experiences of KC patients on perceived service quality received from public facilities.

## Introduction

An estimated 1.3 billion people worldwide are impaired due to cataract, glaucoma, uncorrected refractive errors and corneal diseases as main contributors.^1^ Keratoconus (KC) is a progressive, bilateral but asymmetrical corneal ectasia characterised by high myopia and irregular astigmatism that result from corneal steepening and thinning.^2^ Early studies have presented KC as a noninflammatory condition; however, recently it is confirmed as inflammatory.^3^ Keratoconus onset affects the vision-related quality of life (V-QoL).^4^ At subclinical stages, blurred vision is the commonly reported symptom, although distorted vision occurs as the condition progresses.^4^ Thinning and steepening characterise the various stages of progression and they are accompanied by symptoms such as itchiness, redness and tearing.^3^ In some instances, affected persons experience pain which result from either exposed corneal nerve endings or hydrops at the late stages of progression.^5^

Keratoconus disease is increasingly becoming more prevalent, with an occurrence estimated at 1.38 per 1000 population.^6^ It commonly presents at pubescent years and spans until the fourth decade of life.^5,6^ Some studies reported KC incidences in much older persons.^6^ In terms of gender predilection, there is inconclusive evidence whether the condition is most common in males or females.^6,7^ Higher incidences of KC were realised in countries with warmer climatic conditions than those in colder ones.^6,8^ These variations may be attributed to the environmental exposures in a geographical location and genetic factors of persons in a particular area.^6,8^ Spectacles and soft contact lenses are used to manage KC at subclinical, mild and moderate stages of progression.^9^ Beyond the moderate stages of KC, either corneal or scleral rigid gas permeable (RGP) lenses may be required for vision correction.^9^ Corneas that develop hydrops are treated with hypertonic solution while corneal grafts are conducted when corneas become scarred.^5^ Corneal cross-linking is performed in highly progressive cases of KC.^5^ Besides vision correction, topical antiallergics and pain-relieving eye drops and lubricants are used to manage the impulse of itching, redness and pain.^10^

Irrespective of the lower prevalence and incidence rate in comparison with the other eye diseases, KC affects the V-QoL of much younger people, unlike the other eye diseases which affect the elderly populations.^4,5,6^ Reduced daily activities like those held outdoors, reading, leisure and shopping are restricted due to KC.^11,12^ Further, there is an increase in financial and social impacts on patients with KC.^6,12^

The V-QoL is usually measured through tools such as the National Eye Institute Visual Function Questionnaire (NEI-VFQ 25) tool, which measures the distance and near vision, colour vision tests, dependency, role limitations, mental health and social function, among other variables.^13^ While this accurately measures the outcomes of vision, challenges experienced by patients in accessing such measures are not realised. Upon measurements of the V-QoL, practitioners develop interventions for improvement of these patients’ lives. Patients in a low- to medium-income countries (LMICs), particularly with high socioeconomic inequality like South Africa, are exposed to more factors that exacerbate the effects of these challenges. Over 71% of South Africans are dependent on public eye care,^14^ which is characterised by nonavailability or lack of resources, working space, lack of knowledgeable, experienced and skilled practitioners, and limited access to contact lens services.^15,16,17^ This study aimed to explore the experiences of persons with KC in relation to the care they receive from public sector facilities in the Capricorn District of Limpopo province.

## Research methods and design

### Study design

A descriptive qualitative phenomenology approach was used in the study to explore the lived experiences of patients diagnosed with KC, attending public eye care facilities.

### Study setting

The study was conducted at the public eye care facilities of Capricorn District of Limpopo province, South Africa. The district had eight hospitals, and patients from seven hospitals that granted the study gatekeeper permission were included. In this district, primary eye care was primarily optometrists-led and patients could access eye care services in all eight hospitals. From those that granted gatekeeper permission, six were district-level hospitals, namely Botlokwa, Helen Franz, Lebowakgomo, Pietersburg, Seshego, WF Knobel and Zebediela, while Mankweng was at a tertiary level. District-level hospitals had limited facilities for early detection of KC and could only manage these patients with spectacles. They employed optometrists and ophthalmic nurses only as members of the eye care team. The tertiary hospital had adequate and appropriate facilities to provide comprehensive KC patient management inclusive of contact lenses and additionally employed ophthalmologists in the eye care team who could prescribe antiallergics and pain-relieving eye drops of higher schedules, manage corneal hydrops and perform other surgical procedures on KC patients.

### Selection of patients

The population of the study included all patients diagnosed with KC at any of the targeted public hospitals in the district. Patients with KC who consulted the district-level hospitals and were diagnosed with severe KC not correctable with spectacles hence requiring contact lens fitting or those required management of severe symptoms of KC, hydrops or required surgical intervention by ophthalmologists were sampled. They were regarded to have had sufficient experience of the public service at primary, secondary and/or tertiary level of care hence sampled.

Purposive sampling method was applied to select the sampled patients. Patients were approached when they were attending their appointments at the tertiary hospital between January and March 2021. They were approached by the researcher, face-to-face, just after they had registered for consultation for the day. Children under the age of 12 years were excluded from the study because none of their parents or guardians gave consent for their participation.

### Data collection

Face-to-face, in-depth and semi-structured interviews were conducted to explore the experiences of KC patients attending public eye care facilities on how they were living with the condition, how it was detected, their knowledge of KC, how it had affected their daily living, how they managed to cope with it and the financial burden of living with KC. To elicit the information on the experiences of the patients, an interview guide was used and probing questions were posed so as to get the patients to elaborate on their responses. The interview guide was developed from literature on V-QoL, frequently asked questions and cost of living for KC patients.^12,18,19,20^ Questions on the interview guide are outlined as follows:

- Describe how you have lived with KC since you were young and how it was finally identified.
- How informed are you with KC and how do you manage it in daily living?
- How has the condition affected your daily living?
- How well are you able to do what you always wanted to do as part of your daily routine?
- How has living with KC affected you financially?
- Describe the type of assistance you have received in terms of KC.
- Describe the type of devices you have received to manage KC. How have you been financing the devices and consumables?

Interviews were conducted at the referral hospital, Mankweng Hospital, in a secluded room, in Sepedi language and a few in English, and lasted between 20 min and 35 min. The researcher was fluent in Sepedi and English and this could enable the patients to use either of the two languages they were fluent in. Patients were asked if they will be able to do the interview in English and most opted to use Sepedi. The interviews were recorded and recordings transcribed in the language used for the interview. Due to language differences, intelligent transcribing was used to minimise redundant words and colloquial expressions.^21,22^ This assisted the researchers to provide meaning to words that are not existent in the local language.^21,22^ After transcription, the researcher translated all transcripts into the English language. An English language expert who was also fluent in Sepedi conducted verification of the transcripts, and their accuracy was verified by firstly checking the original audio recordings against the transcript and, secondly, by checking and correcting any errors in translation between the Sepedi and English transcripts.^23^

### Data analysis

Each transcript was analysed individually. NVivo version 12 was used to develop and group codes into themes. Codes were inductively generated from data, grouped into various themes in line with the Braun and Clarke’s approach of thematic analysis.^23^ This followed a step-by-step approach where the researcher read through the transcripts so as to be familiarised with data. These were followed by identification of patterns and development of codes, where similar or related codes were grouped into categories of themes for interpretation.^24^

### Study rigour

The study protocol was prepared and submitted for ethical clearance so as to ensure that the research process observed the ethical guidelines of the University of KwaZulu-Natal (UKZN) Biomedical Research Ethics Committee (BREC).^17^ Pilot interviews were conducted to shape the interview tool so as to eliminate any leading questions and those with potential for harm. Two pilot interviews were conducted and the researcher made necessary revisions on the interview process. The data from this pilot interviews were not included in the analysis.^25^ The researchers applied an iterative process throughout the analysis to confirm the meaning of codes, categories and themes.^17^ Triangulation of the transcriptions, memo and field notes were performed to strengthen understanding and to construct actual meanings of phrases.^26^

### Ethical considerations

An application for full ethical approval was made to the Biomedical Research Ethics Committee of the University of KwaZulu-Natal and ethics approval was received on 08 May 2020. The ethics approval number is BREC/000.01223/2020. Permission to conduct the study was also obtained from the Limpopo Province Department of Health (LP-202005-002).

Patients were given invitation letters to participate in the study upon their arrival and registration at the hospital. This letter informed them of voluntary participation and that they could withdraw from the study at any time during the data collection process. The letter further informed patients about consent of participation which stated that only those who met the criteria and also gave consent to participate would be included in the study.

## Results

Sixteen patients were selected, of which nine were male and seven female. Of the 16, five were learners at various grades in school, from Grade 9 to Grade 11. Five were neither employed nor in school, four were attending tertiary institutions, one was employed and another self-employed (see Table 1). Patients spoke Sepedi as their native language, although most had understanding of English or used English as a language of instruction in universities.

**TABLE 1:** Characteristics of patients.

Participant	Age (years)	Gender	Occupation
1	23	Male	University student
2	37	Male	Self-employed architectural technologist
3	35	Female	Unemployed
4	16	Male	Grade 11 learner
5	23	Male	College student
6	15	Female	Grade 9 learner
7	34	Female	Unemployed
8	20	Male	Grade 11 learner
9	25	Male	University student
10	26	Female	Unemployed
11	38	Female	Unemployed
12	15	Female	Grade 9 learner
13	26	Female	College student
14	23	Male	University student
15	42	Male	General worker
16	30	Female	Unemployed

### Theme 1: First experience of keratoconus presentation

#### Subtheme: Condition started at early age and got worse as patients grew older

Many patients realised that they had problems with their vision from a young age, which became worse as they grew older. Most patients reported that the problems started when they were at school before their teen ages:

‘I had problems since I was young. It got more difficult as I grew up.’ (Participant 1, male, 23 years old)

‘I had problems [*of not seeing well*] since I was in grade 3 and started wearing spectacles when I was grade 4.’ (Participant 2, male, 37 years old)

Only one participant reported to have realised the problem when they were older:

‘I started realising the problem at age of 30 years. I could no longer drive comfortably and could hardly see much at night.’ (Participant 3, female, 35 years old)

**TABLE 2:** Themes and subthemes developed from patients’ interviews.

Theme	1. First experience of KC presentation	2. Knowledge about KC	3. Challenges experienced when performing daily activities	4. Experiences of bullying by learners and negligence by practitioners	5. Expenses associated with living with keratoconus	6. Coping mechanisms
**Subthemes**	Condition started at early age and got worse as patients grew older	Patients did not receive adequate information about their condition	Learners had difficulty seeing the writings on the board at school	Some learners experienced bullying from teachers and fellow learners	Most patients were from low-income families and some were social grant recipients	Learners relied on friends and peers in class for coping with the requirements of class activities
	Patients had history of allergies	Patients were either misinformed or ill-informed	Learners had to do much more work to compensate for the visual inefficiencies	Few patients experienced negligence from practitioners	Patients had to visit the hospitals more frequently, which was costly in terms of transport and hospital fees	Some learners used multimodal learning platforms to compensate for their vision inefficiencies
	Teachers at schools played a critical role in detecting vision problems	Language and literacy were barriers to receiving relevant information	Patients worked at a slower pace and a shorter distance than other average persons	-	Patients incurred high costs when using private practitioners where public service was deficient	Most of the patients relied on family support to cope on a daily basis
	Learners used various methods to self-diagnose their vision deterioration	-	Some learners dropped out of school and some adults could not get employment	-	Patients wearing contact lenses acquired additional costs for purchasing contact lens solutions and consumables	-
	-	-	Patients reported that they depended more on other persons on daily basis	-	-	-

KC, keratoconus.

#### Subtheme: History of allergies

In many of the cases, patients reported to have had allergies which were followed by the deterioration of their vision. Many of the patients were taking medication on a frequent basis to manage their long-standing allergies of the eyes:

‘I grew up consulting and I was told I had allergies. I realised I had problems with my vision when I was ten years old. My eyes were itchy and I could not see. My eyes were also teary. Vision got worse in 2013 to 2014, and in 2015 I could not see at all.’ (Participant 4, male, 16 years old)

‘I have been taking medication since 2005.’ (Participant 3, female, 35 years old)

#### Subtheme: Teachers at schools played a critical role in detecting vision problems

Teachers made initial observations that some of the patients had vision problems. They alerted their parents and recommended that the patients go consult for eye examination:

‘My teacher then, realised that when she gave some work to do that I copied from peers. Besides that, my work was not tidy. I also took a longer time to copy from the board. In some instances, I just left black spaces. Then she recommended that I go to hospital for treatment.’ (Participant 3, female 35 years old)

‘I was screened at school and had my first pair of spectacles in 2012. I was diagnosed [*with keratoconus*] in 2016…’ (Participant 5, male, 23 years old)

#### Subtheme: Learners used various methods to self-diagnose

One of the learners reported that he realised that he was having a problem when his peers could see what he could not see. He consistently compared himself but realised his shortfall:

‘When I was in grade 7, I started noting that people [*peers*] can see things that I cannot see.’ (Participant 6, female, 15 years old)

### Theme 2: Knowledge about keratoconus

#### Subtheme: Not receiving adequate information

Several patients felt they were not provided with adequate information so as to know about their condition. They lived with allergies for a long time and their vision deteriorated in the process. They, however, got to be diagnosed and informed of KC only recently. Some feel they were undermined by parents then, because they were perceived as being younger. Regarding the inadequate information, they reported that they could have paid more attention and used other avenues to get help had they been better informed:

‘I went to many hospitals, even the train [*Phelophepa Health train*]. I still didn’t know that I have keratoconus.’ (Participant 6, female, 15 years old)

‘I was still young and I was not provided with relevant information. I was forced to take medication by parents but did not understand why that was the case. I was undermined because I was young. I only got informed that I have KC last year. If the condition was explained to me, I could have paid more attention to the condition.’ (Participant 7, female, 37 years old)

Patients with higher literacy level and exposure to information platforms like internet could describe the conditions. They included all university students and a few high school learners that reported to having been using the internet. They reported that they did their own research by searching on the internet for relevant information:

‘I searched the internet to understand more things about keratoconus.’ (Participant 8, male, 20 years old)

Some still reported not having had much information about KC and how it could be managed:

‘They never told me about my condition. I just know that I cannot see. One eye does not see anything at all … I was never told of contact lenses. I was just referred here. They need to check my eyes.’ (Participant 2, male, 37 years old)

#### Subtheme: Patients misinformed or ill-informed

Some patients consulted private optometrists and ophthalmologists who informed them that they needed a donation of the cornea and corneal surgery to help them see. Others were told that they needed corneal cross-linking treatment to stop the condition from getting worse. No other management options were suggested at that stage. They got discouraged to seek further help because they could not afford the said surgical procedures:

‘I was taken to a doctor who diagnosed me but told me that I would need a donor [*corneal donation*]. However, I could not afford the service.’ (Participant 9, male, 25 years old)

#### Subtheme: Language and literacy barrier

Most patients could not clearly explain what they understood KC to be. Patients reported that practitioners used simple layman’s language which could not provide adequate description for understanding the condition. When asked to provide their description of the condition, neither an ordinary person nor another practitioner who did not have prior knowledge of their condition could put into context that they were describing KC. One participant explained a corneal hydrops as a ‘crack’ that developed on the eye which caused leakages on to the eye. Another described the shape of the eye as that of a ‘rugby ball’ but could not provide any other description to provide the context for understanding:

‘I was told I have cracks and my eyes were leaking. And it is painful.’ (Participant 9, male, 25 years old)

‘They said my cornea is like a rugby ball.’ (Participant 10, female, 26 years old)

### Theme 3: Challenges experienced when performing daily activities

Many patients reported that they had many challenges resulting from their inability to see.

#### Subtheme: Learners had difficulty seeing at the board

Learners reported inconveniences in their lives caused by the inability to see clearly. They reported that they had to change their sitting arrangements in the classrooms to compensate for their inability to see the writings on the board in front:

‘I have to go early to school such that I book a table in front … if I arrive late, I will have to sit at the back. Then I squint my eyes and it becomes painful.’ (Participant 10, female, 26 years old)

A few reported that they could not see even though they changed seats to be closer to the board. These learners only managed by asking to copy work of their peers so as to be on par with the rest of the learners:

‘When the teacher wrote on the board, I could not see what they were writing. I could only hear.’ (Participant 6, female, 15 years old)

#### Subtheme: Doing extra to compensate for the visual inefficiencies

Learners felt that they had to do more than their peers so as to be on par with them in school. They had to arrive at school much earlier than their peers to book seats so that they could sit in front. Some could not see the writings on the board when the teacher was in class:

‘I have to go early to school such that I book a table in front.’ (Participant 10, female, 26 years old)

They had to search for online videos to supplement what they heard in class so that they could gain adequate knowledge on the topics of interest. Due to the inability to see clearly, several patients reported that they had to listen much more attentively so as to be able to capture the topics covered for the day.

#### Subtheme: Patients worked at a slower pace and a shorter distance than other average persons

More patients reported that the speed at which they completed their tasks was slower than that of their peers because of their working distance. They had to lean over very close to the material they are reading so as to be able to see:

‘I realised that my eyes are having a problem in 2009 when I was in college. In 2011, it got worse. I tried sitting in front but it was still difficult. I managed to complete my college diploma with difficulty though. When doing computer studies, I had to hold my worksheet in one hand [*to bring it close to my eyes*] and type [*on the keyboard*] with the other hand. I worked at very close distance. I did Business Management until N6. However, I could not do internship or in-service training because I could not see anything.’ (Participant 11, female, 38 years old)

#### Subtheme: Patients dropped out of school and others could not get employment

Several patients dropped out of school or university and some reported that they could not secure employment even if they have qualifications because they could not see. Some remain unemployed:

‘I repeated my first year at varsity. Then, I got expelled for failing [*for the second time*] to achieve a set number of modules at university. Last year, I spend the entire year without studying.’ (Participant 1, male, 23 years old)

‘I failed my matric because I could not see any more on the board.’ (Participant 11, female, 38 years old)

‘I could not pursue my education because of this condition. I failed matric because it was difficult to see.’ (Participant 11, female, 38 years old)

One of the patients took a job as a domestic worker (helper) but was dismissed because she could not clean the floors properly and she consistently dropped and shattered drinking glasses and other dishes:

‘I tried to clean but could not see if the floor was indeed clean. I also broke the glasses [*drinking glasses*] daily when washing them.’ (Participant 3, female, 35 years old)

#### Subtheme: Dependence on other persons

Patients felt that having KC compromised them because they had to be dependent on other people in day-to-day living. Several patients reported that they could not do house chores efficiently:

‘I cannot even see if the floors are clean or not.’ (Participant 9, male, 25 years old)

One participant reported that she had to unwillingly share on issues that she regarded too personal to share with any person mainly to get assisted. Sometimes people had to enter her personal space in the bedroom and go through her personal items when having to help her. She felt compromised.

### Theme 4: Experiences of bullying by learners

#### Subtheme: Bullying by teachers and learners

Learners reported that they received support from their teachers and fellow learners at schools. Teachers allowed them to copy work from their peers so as to supplement what they could not see on the board during class proceedings. Some teachers continuously asked the affected learners to update them on things they needed assistance with and they constantly provided support.

Teachers would even make provision for the learners to sit on the front benches so that they could see the writings on the board. Others report to parents when learners were struggling at school.

There were, however, isolated incidences of bullying from some teachers and learners. Some teachers used derogatory language on the learners because of their inability to see the writings on the board and in the process some of learners or peers would be laughing:

‘Teachers do not give me problems when I have to copy from my friend because they know I have a problem. But there is this other ma’am [*female teacher*] who bullies me. She would snap and shout at me calling me names because I have to squint my eyes to be able to see and avoid excessive light. My mother started paying attention to my condition only after the school called home.’ (Participant 12, female, 15 years old)

#### Subtheme: Negligence by practitioners

In an isolated incident, one of the patients reported that he felt he was bullied when attending at one of the hospitals. He attended the hospital on numerous times and was inaccurately diagnosed but rather told he had allergies. The practitioners were very impatient and not willing to attend to him further:

‘I went to the hospital but they rejected me. They said I just had bad allergies but I am fine.’ (Participant 4, male, 16 years old)

### Theme 5: Expenses associated with living with keratoconus

Several patients reported that they could not afford costs that come with living with KC. All patients depended on the family for financial support.

#### Subtheme: Low-income families and social grant recipients

Although some financial support was provided from family members, the financial burden proved to be difficult, especially when there were inconsistencies with personal or household income. This resulted in patients being dependent on other people for financial support or using their social grant funds. The social grant money would be used to fund the costs for either travelling to hospital or for consultations. One of the patients who is an unemployed mother used money from her child’s social grant to travel to the hospital for treatment:

‘I have a child who receive child support grant. Each time I know I have to consult, I will put away R80.00 for myself from that. Then it helps me. There is also transport that I have to pay also. It is R60.00 from the hospital to where I stay …’ (Participant 11, female, 38 years old)

‘I am not employed nor at school … my father pay for the costs and sometimes my sister does.’ (Participant 16, female, 30 years old)

Some forfeited their appointment on a number of occasions due to unavailability of funds for either transport or consultation.

‘… [*S*]ometimes I forfeit my consultations due to non-availability of funds when parents are not home. They take long to come back from work, they work in Gauteng.’ (Participant 5, male, 23 years old)

#### Subtheme: Frequency of hospital visits

Most patients with long-standing allergies either consulted or collected medication from hospitals on a frequent basis. They had to consult with the ophthalmologists at a tertiary hospital at least once in a month, a quarter or a six month period depending on the severity of their condition. Some received their prescriptions but had to collect the medication monthly at a local hospital. This drove costs higher because a number of them stayed far from the tertiary hospital. They had to pay each time they attend at any of the hospitals and had additional costs of travelling:

‘My parents pay for my daily travelling of R50.00 each time I consult.’ (Participant 5, male, 23 years old)

‘There is also transport that I have to pay also. It is R60.00 from the hospital to where I stay.’ (Participant 7, female, 34 years old)

‘I consult monthly at the base hospital. I pay R40.00 for transport and R70.00 for consultation.’ (Participant 10, female, 26 years old)

‘Each and every time I come (to consult), a pay for a file. I pay R80.00 for consultation.’ (Participant 7, female, 34 years old)

#### Subtheme: Patients incurred high costs when using private practitioners where public service was deficient

In some instances, the hospitals did not provide spectacles or patients had to wait for extended period of a year or more to receive their spectacles. Some patients reported that they resorted to using private practitioners to get spectacles.

‘[*W*]e paid between R2900.000 and R3500.00 once every two years …’ (Participant 1, male, 23 years old)

‘My father pays for spectacles… we don’t have medical aid. He pays cash. He paid R3000.00 for my spectacles …’ (Participant 12, female, 15 years old)

#### Subtheme: Patients incurred additional costs for contact lens solutions and consumables

Besides consultations, travelling and spectacles, contact lens solutions and consumables required for contact lens wearers were an additional cost. They found this expensive:

‘I have to buy my contact lens solutions. I use Boston and other multipurpose solutions. They are expensive though. I pay about R250.00 for them monthly.’ (Participant 2, male, 37 years old)

### Theme 6: Coping mechanisms

#### Subtheme: Reliance on friends and peers in class

Several patients, especially learners and students, reported that they had to develop stronger relationships with peers so that they could benefit from them in class. They got additional information on work that they could not copy from the board on a daily basis. This, according to one of the patients, was highly beneficial, and when they had to separate with these friends after matric, they were left destitute. He used this coping mechanism but struggled when he went to university where his friends were no longer there to assist. In addition, the classes (number of students in a class) were larger and it was difficult to always get to class early to occupy front seats. Besides, everybody has to pay attention and therefore he could not continuously disturb them when in class:

‘I use to sit with my friend even in high school, when he wrote on his book then I would copy from him. But when I went to tertiary we got separated. Then I started having problems.’ (Participant 1, male, 23 years old)

#### Subtheme: Use of multimodal learning

Several patients indicated that it was important to use additional online video and audio lectures to supplement the notes collected from their class. They would view or listen online and download those possible so that they could understand the topics of interest fully. This, however, put a lot of pressure on the patients because they took more time to be on par with their peers:

‘[*L*]et’s say they introduce a digestive system, when I get home, I search for it on the internet. I use the videos to enrich my knowledge.’ (Participant 4, male, 16 years old)

One participant dropped out of varsity because it mostly used venue-based learning. Then he failed because he could not cope. But he managed to register in another institution that moved to multimodal learning after the coronavirus disease 2019 (COVID-19) pandemic hit. He is coping well now because he does most of the learning on a computer screen. He can play and replay the videos of the lectures.

#### Subtheme: Family support

Patients reported that they received support from their families. Their parents and siblings were of great assistance with activities they engaged in on a daily basis. They reported that they also got emotional support especially from their parents. However, several patients indicated that when their family structure was compromised, they were severely affected. A number of patients had to discontinue their treatment because their parents migrated from home to seek employment in the urban areas. In another instance, a participant who lived with his grandmother could not be taken for regular consultations and collection of their medication. This only resumed when the parents moved back home after a number of years.

‘[*M*]y parents left went to Gauteng work, no one could take me for treatment and I had to stop taking treatment.’ (Participant 8, male, 20 years old)

Besides receiving assistance with some of the physical activities, all patients reported that their families supported them financially. This was also the case with those that were in some form of employment.

‘My mom bought me a router to connect to the internet for searching instructional videos…’ (Participant 4, male, 16 years old)

‘My father gives me money to pay for the consultation.’ (Participant 9, male, 25 years old)

‘My mom helps me. She sometimes cover my costs …’ (Participant 2, male, 37 years old)

Other family members and friends played a critical support role to the parents in order to help.

‘I told my grandmother. She forced my mother to take me to a doctor. My mother’s employer pays for my medication. She [*mom’s employer*], took me for a consultation and they realised that my left eye is very weak and also that I needed spectacles.’ (Participant 12, female, 15 years old)

## Discussion

The study aimed to explore the lived experiences of persons affected by KC that attend the Capricorn District of Limpopo Province. Unlike the V-QoL outcomes presented by authors such as Kymes et al.^4^ on the topic, this study aimed to describe the experiences of patients on the challenges of living with KC on a daily basis as well as the type of care and support they receive. Patients diagnosed with KC reported to have developed gradual vision loss which worsened as they grew older, which was consistent with literature. Keratoconus is a progressive condition characterised by ocular discomforts and gradual vision loss as a result of corneal steepening and thinning, which cause high myopia and irregular astigmatism.^4,27^ If not managed, it may result in significant visual impairment with negative health outcomes and poor quality of life.^28^ Persisting symptoms including those that are allergy-related compromise the quality of life (QoL).^29,30^

Key determinants for diagnosing KC are corneal steepening and thinning,^31^ which are detected when a patient consults with an eye care practitioner. In a normal patient examination, corneal thinning and steepening may not be detected unless a keratometry, corneal topography and/or ocular coherence tomography are performed. In the absence of these procedures, patients may experience symptoms and signs that relate to KC, suggesting its onset. Patients in this study reported experiencing gradual vision loss and presence of itchiness that required constant rubbing. When these are experienced, KC may be suspected and the affected persons should consult for KC screening. From the findings in the study, many patients were not working and could not afford regular consultation at a public facility; hence, they did not receive timely service.

Vision problems are commonly detected through programmes such as school-based vision screening.^32^ These programmes are sometimes deficient in detecting certain conditions like KC due to availability of equipment and perhaps lack of expertise if ophthalmic nurses and optometrists are not involved during the school visit.^33^ Teachers are still able to identify children with visual impairments when they bump into objects, are unable to focus on objects or follow them, excessively rub and squint their eyes, have trouble reading and participating in class and are unable to see distant objects.^34^ Many of the patients reported that their teachers and schools played a critical role in detecting their visual impairment which was later diagnosed as KC. One of the patients self-identified to having vision problems which, according to Willings,^35^ is beneficial to the patients to communicate their needs early. Compulsory vision screenings may be implemented in schools at entry levels of the foundation, intermediate and senior phase to enhance early detection of abnormalities of the vision and eye health. In addition, vision and eye health education programmes can be implemented in schools to provide basic skills to all teachers to enable them to easily identify children with vision problems. These initiatives will contribute to early detection of KC, which is an effective approach to arrest KC progression and rehabilitate the loss of vision^16^ with appropriate correction devices, including spectacles at early stages and also contact lenses as the condition progresses.^9,36^ If KC is detected early, symptoms that contribute to constant rubbing and those of pain may be managed with medication, consequently slowing down the rate of progression.^37^

Patients in this study reported to have received limited information regarding KC and that their knowledge of this condition was inadequate. Similar findings were realised in a Swiss-based population where only one-third had minimal keratoconus knowledge (MKK) and presumably the other two-thirds did not have.^38^ Baenninger et al.^38^ define some aspects which any patients should know about KC, which include being able to describe or define KC, its risk factors and triggers, knowledge of symptoms and consequences of untreated KC and also treatment options not limited to spectacles, contact lenses, corneal cross-linking and corneal surgery. Most patients in this study could neither provide a definition or description of KC nor indicate its risk factors and triggers. They were aware of their vision deterioration and the symptoms but they could not relate most of them to KC. In terms of treatment options, most patients were told that they were consulting to be fitted with contact lenses to manage KC but could not provide additional information. Unlike the study by Baenninger et al.,^38^ findings in this study were possibly exaggerated by the poor health literacy and language barriers. This information should be provided to patients by their practitioners after they are diagnosed or through awareness campaigns. Further, after diagnosis of KC, patients may be referred to read up or get more information so as to know about the condition, thereby improving the health literacy. Patients who had access to information platforms such as internet had better knowledge of KC, suggesting that they accessed and read on KC. Patients in these facilities were detected at very late stages when signs such as Munson’s sign were visible due to lack of knowledge, skills and basic equipment.^39^ In addition, only a half (54.2%) of the optometrists in these facilities self-reported to have adequate knowledge and skills to screen, examine, diagnose and manage KC.^16^ Poor knowledge and skills of practitioners could have contributed to poor patient education and consequently poor health literacy,^40^ hence the lack of knowledge of KC as described in the study by Baenninger et al.^38^

The description of KC by patients using words like the ‘eye having cracks’ or the eye having a ‘rugby ball shape’ insinuates limitations in healthcare vocabulary, especially for native speakers who need to provide descriptions of the eye conditions. These patients needed to relate the words to the native language speakers although the words used not formalised or standardised for describing this condition. The challenge in this case is also about the lack of development of the indigenous language to better explain the conditions better understood in English.

It is therefore necessary to consider translation of such words to the local languages using organisations such as Pan South African Language Board (PanSALB). This may be best achieved through a collaboration of optometry training institutions and PanSALB.

The onset of KC poses serious challenges where those affected are unable to perform their usual daily activities.^13^ Keratoconus is a bilateral but asymmetrical condition; therefore, many patients usually seek consultation when vision in the better eye has deteriorated, and by that time they would have had significant vision reduction in the worst eye.^39^ In this study, many patients reported disruption in their normal performance of daily activities as they could no longer see well the writings on the board in school, some had to work more than the average learner so as to be on par with peers, some worked slower than they used to and others had to drop out of school. This affected their QoL and forced them to have some dependence on other people to cope with daily activities. A major challenge with KC is that although it has lower prevalence, it affects young adults and it also has severe negative consequences on their quality of life from young age.^13^ The Collaborative Longitudinal Evaluation of Keratoconus (CLEK)^12^ study reported challenges of mental health, ocular pain, inability or reduced ability to drive, and dependency in the patients. Efforts should be put forth to strengthen the systems at all levels of eye care, including primary, secondary and tertiary, so as to detect KC early and initiate treatment for better visual and eye health outcomes to affected patients. These should include a holistic approach to eye care, including school eye health programmes and use of community clinics as centres of care.

Some patients in this study reported victimisation, including name calling, by their teachers or peers. Bullying of children with visual impairments, including KC in this case, is not an isolated incident or a new thing. Pinquart and Pfeiffer^41^ in their study found high levels of peer victimisation and relational victimisation in partially sighted and blind adolescents than in sighted adolescents. Mainstream schools are less prepared to accommodate children with disabilities like visual impairments. Peer support, peer and teacher education are necessary to curb the incidences of bullying of any form and to promote inclusive education by creating an enabling environment for children with visual impairments like those with KC.^41,42,43,44^

Patients reported various financial challenges of living with KC, which included transportation and consultation fees with the frequent consultations. Additionally, some had to buy contact lens solutions and consumables for contact lens use. Some patients paid higher out-of-pocket amounts to get optical devices like spectacles because there were delays from the public eye care facilities. Iskandarsyah et al.^45^ suggest that transport costs and consulting fees, cost of spectacles and medication are barriers to healthcare. The findings in this study agree with those of Iskandarsyah et al.^45^ There is a need for adequate planning in view of strengthening health systems for effective governance and leadership, a responsive, competent and productive eye care workforce, an accessible, affordable and equitable service provision, and a fully equipped and technologically advanced infrastructure by hospitals and government to reduce waiting times and increase access to spectacles and contact lenses. Increasing access to contact lens fitting across all hospitals may also reduce the number of referrals to the tertiary hospitals, hence reducing costs. The majority of persons (62.7%) in Capricorn District rely on the public service and they therefore suffer in case some of the services are not fully provided or if there are waiting times before patients can receive a service and/or a device.

A few patients in this study reported to have developed skills to adopt online learning platforms to cope with the demand of their study programmes and compensate for their deficiencies in vision when they are at institutions of learning, including schools and universities. They also stated that they arrived much earlier at the classrooms in order to pick the best spots to sit, and also not to leave classrooms, unless pertinent, so as to maintain their occupied spots to sit in during the day. With the onset of visual impairment, patients learn new skills to cope with the new challenges. They set new goals and integrate the use of vision correction devices and medication for ocular health in their routine.^46^ Patients come up with initiatives similar to those mentioned when they start accepting their impairment.^46^ Emotional, social, practical and physical support is, however, required for the visually impaired because it highly benefits affected persons and enhances their faster recovery or acceptance.^46^ Patients in this study indicated receiving family support and support from peers and teachers at school. Use of therapists for psychosocial support should be explored to assist those that may be struggling to get to terms with their impairments.

### Strength and limitations of the study

A qualitative research approach was used in this study in order to allow an exploration of the patients’ experiences of daily life. They were able to share their experiences and narrate them from their own perspectives. This strength of the study enabled this study to complement findings in previous studies which only included variables like visual acuity, contract sensitivity and colour vision among others. Besides having KC, patients in this study were affected by socioeconomic challenges common within LMICs, which is not common to usual patients from high-income countries. Besides the patients, the design of the study provided a context of the environment patients live in, which exacerbates their experience of challenges. Interviews were conducted in the language better understood by the patients; however, there could have been limitations with the transcription and translation of the interview recordings. This was, however, overcome by the researcher’s fluency in both English and Sepedi and with the help of a language expert who was also fluent in both languages for verification of the transcriptions.

## Conclusion

The study presented the experiences of patients and the effect of visual impairment brought by KC. Existing literature generally focused on the clinical findings in KC patients and their effect on daily lives, based on quantitative research approaches. This exploration allowed patients to narrate their daily experiences. Patients reported to have experienced KC from a young age where onset of gradual vision loss and itchiness were noted. The study further found that patients had limited knowledge of KC, and literacy and language barriers exacerbated this knowledge gap. Educational programmes to patients need to be developed to help them self-identify whether they are at risk of or are developing eye care conditions. School-based programmes targeting teachers may also be developed so as to help teachers identify children that have visual inefficiencies. Collaborations with organisations such as PanSALB are also necessary to develop local languages so as to accommodate the translation of names of conditions from English or other languages of origin. There were daily challenges in performing regular tasks; however, those diagnosed developed various coping mechanisms and relied heavily on family and peers for support. Families need to be involved in the care of their family members with KC so as to broaden the potential support. Schools may also be developed to accommodate children with visual disabilities. The patients found living with KC more costly because even when they used public eye care service which is perceived to be more cost-effective, there were higher out-of-pocket payments. They incurred costs of frequent travels to hospitals; they had to buy consumables and some bought optical devices when the hospitals were unable to. Proper planning is necessary to help public facilities cope with the onset or development of newer eye conditions or the increase in their incidences.
